# Aconiti Lateralis Preparata Radix Activates the Proliferation of Mouse Bone Marrow Mesenchymal Stem Cells and Induces Osteogenic Lineage Differentiation through the Bone Morphogenetic Protein-2/Smad-Dependent Runx2 Pathway

**DOI:** 10.1155/2013/586741

**Published:** 2013-07-29

**Authors:** Do Rim Kim, Ha Young Kim, Jae Kwang Park, Seong Kyu Park, Mun Seog Chang

**Affiliations:** Department of Prescriptionology, College of Korean Medicine, Kyung Hee University, 26 Kyunghee Dae-ro, Dongdaemun-gu, Seoul 130-701, Republic of Korea

## Abstract

Mesenchymal stem cells have the capacity for self-renewal and under appropriate stimulation give rise to osteogenic, adipogenic, and chondrogenic lineages. To advance the clinical use of stem cell therapy, such as stem cell transplantation, it is important to find substances that promote endogenous stem cell proliferation and differentiation. We investigated whether medicinal herbs have the potential to promote stem cell proliferation and differentiation, using a cell cycle analysis and differentiation assay. We found that Aconiti Lateralis Preparata Radix (ALR) promoted the proliferation rate of mouse bone marrow mesenchymal stem cells (mBMMSCs) up to 122.24% compared to untreated cells. Fluorescence-activated cell sorter analysis showed that the percentage of cells in the G2/M phase increased to 17.33% in ALR-treated cells compared to 5.65% in normal cells. Signaling pathway analysis indicated that this was mediated through the extracellular signal-regulated kinase 1/2 pathway. A differentiation assay showed that ALR induced differentiation of mBMMSCs into an osteogenic lineage 2 weeks after treatment, whereas traditional osteogenic induction medium treatment did not promote differentiation for 3 weeks. This osteogenic differentiation was signaled by the bone morphogenetic protein-2/Smad-dependent Runx2 pathway. We found that ALR could promote mBMMSC proliferation and differentiation into the osteogenic lineage.

## 1. Introduction

Adult stem cells are involved in the repair of tissue and in maintaining a balance between stem cell and differentiated cell populations by asymmetrical cell division [1]. Although adult stem cell populations are found in most adult tissue, the bone marrow is an ideal source of stem cells because it is easily accessible and can be used for cell and gene therapy [1, 2]. Mesenchymal stem cells (MSCs) are derived from bone marrow and represent a heterogeneous cell population of spindle-shaped cells that are characteristically adherent to plastic in culture [3]. MSCs have been isolated and cultured from many species including mice, rats, cats, dogs, rabbits, pigs, and baboons, albeit with varying success and with the expression of varied surface markers. MSCs have also been found to give rise to differentiated stromal cells belonging to the osteogenic, chondrogenic, adipogenic, myogenic, and fibroblastic lineages [4].

Some cytokine and growth factors involved in mitogen-activated protein kinase (MAPK) signaling are known to enhance MSC proliferation. Platelet-derived growth factor (PDGF) and fibroblast growth factor 2 (FGF2) have been known to enhance proliferation through c-Jun N-terminal kinase (JNK) signaling [5]. In addition, basic fibroblast growth factor (bFGF) is known to stimulate human bone marrow mesenchymal stem cell proliferation via extracellular signal-regulated kinase 1/2 pathway (ERK1/2). However, neither PDGF-BB nor bFGF-induced proliferation affects the osteogenic differentiation potential [6].

Cytokines and growth factors have important roles in stages ranging from self-renewal to differentiation; however, the molecular mechanisms involved in these processes are still largely unknown and have practical limitations [7]. Furthermore, little is known about the involvement of medicinal herbs in the proliferation and differentiation of bone marrow mesenchymal stem cells. The involvement of a signal pathway that regulates proliferation and differentiation of MSCs by medicinal herbs in bone marrow has not been reported. A recent study has reported that Aconiti Ciliare Tuber extract promotes hair follicle morphogenesis by the activation of Wnt/*β*-catenin signaling to stimulate bulge stem cells [8].

The aim of the present study was to examine the possibility of ALR inducing proliferation and/or differentiation of mouse bone marrow mesenchymal stem cells (mBMMSCs) to replace the function of growth factors and cytokines and to elucidate the signaling pathways involved in these processes. To identify the effects of ALR on MSCs, we performed cell- and cell cycle-analysis, immunoblotting, immunohistochemistry, and differentiation assays.

## 2. Materials and Methods

### 2.1. Preparation of ALR Extract

ALR was purchased from Wonk Wang Herbal Drug Co. Ltd. (Seoul, Korea). We boiled 250 g of dried ALR in 5 L of water for 2 h at 100°C. The suspension was filtered and evaporated under reduced pressure. The yield of extraction was 17.1% (42.8 g). The filtrates were lyophilized and kept at 4°C. Before each experiment, dried extracts were dissolved in distilled deionized water (Millipore, Bedford, MA, USA) and vortexed for 2 min at room temperature. 

### 2.2. Analysis ALR Sample Using Liquid Chromatography-Tandem Mass Spectrometry (LC-MS/MS)

Stock solution of reference standards, aconitine, and benzoylaconine were prepared in 100% methanol at a concentration of 1 mg/mL. The working solutions for MS analysis were prepared in 100% methanol at a concentration of 1000 ng/mL. For standard curve, six calibration standards (at 10, 50, 100, 200, 500, and 1000 ng/mL) were analyzed. Lyophilized ALR extract was dissolved as 100 *μ*g/mL in 100% methanol. The solution was separated by centrifugation at 2500 rpm for 15 min. The supernatant clear extract was filtered through 0.45 *μ*m membrane filter prior to LC/MS/MS determination. Chromatography was performed on a liquid chromatography system (Agilent Technologies, CA, USA). A 500 *μ*L aliquot of diluted ALR was injected into a 100 mm × 2.1 mm Atlantis DC18 column (Waters, USA). Mobile phases A and B consisted of 0.1% aqueous formic acid and acetonitrile, respectively. The elution followed a linear gradient of 5–95% for 20 min at a flow rate of 0.3 mL/min. QTRAP 3200 mass spectrometer system (ABSCIEX, MA, USA) operated in positive ionization modes was used in this study and processed multiple reactions monitoring (MRM) scanning. Mass spectrometer tuning parameters were optimized for each analyte by injecting a standard solution (0.001 mg/mL) and validated with several flow rates ranging from 0.1 to 0.5 mL/min. The mobile phase contains acetonitrile in water, adding 0.1% (v/v) formic acid. Under the optimized condition for positive mode, nitrogen was used as drying gas, 10 Lmin^−1^, and nebulizer gas, 50 psi, while the voltage of ion spray in source was set at 5000 V and the gas temperature was 400°C.

### 2.3. Isolation of BMMSCs

Mouse stromal cells were isolated according to the methods of Nadri et al. [9], with some modification ion. Bone marrow cells were obtained by flushing the femurs and tibias of 8- to 12-week-old male C57BL/6 mice with Dulbecco's modified Eagle's medium (DMEM) (Gibco-BRL, Grand Island, NY, USA) supplemented with 15% fetal bovine serum (FBS, Gibco-BRL, Grand Island, NY, USA), 100 U/mL penicillin, and 100 *μ*g/mL streptomycin. Single-cell suspensions were prepared from clumps of bone marrow by resuspending the cells using a mounted 26-gauge needle syringe and passing them through a 70 *μ*m cell strainer (Falcon, Becton Dickinson and Company, Heidelberg, Germany). The cells were cultured in DMEM with 15% FBS, 100 U/mL penicillin, and 100 *μ*g/mL streptomycin on 0.1% gelatin-coated 10 cm dishes at 37°C in a humidified atmosphere of 5% CO_2_. After 3 days, nonadherent cells were removed, and adherent cells were expanded until 90% confluent (6 to 7 days). All animal procedures were performed in accordance with institutional guidelines.

### 2.4. Sorting of Sca-1-Positive and CD45-Negative BMMSCs Using Magnetic-Activated Cell Sorting (MACS)

Enrichment of Sca-1-positive (+) BMMSCs was achieved by an MACS system (Miltenyi Biotec GmbH, Bergisch Gladbach, Germany). Following the third sorting passage, BMMSCs were incubated with phycoerythrin-(PE) conjugated anti-CD45 antibody (Miltenyi Biotec GmbH, Bergisch Gladbach, Germany) for 10 min at 4°C and washed with MACS buffer (phosphate-buffered saline (PBS) supplemented with 0.5% BSA and 2 mM EDTA). Anti-PE microbeads (Miltenyi Biotec GmbH, Bergisch Gladbach, Germany) were then incubated for 10 min at 4°C and washed once again with MACS buffer. The samples were passed through an MACS column in a Miltenyi magnet. The CD45 (−) BMMSCs were incubated with fluorescein isothiocyanate-(FITC) conjugated anti-Sca-1 antibody (Miltenyi Biotec) for 10 min at 4°C and then washed with MACS buffer. Anti-FITC microbeads (Miltenyi Biotec) were then incubated for 10 min at 4°C and again washed with MACS buffer. Samples were passed through an MACS column in a Miltenyi magnet and the Sca-1 (+) BMMSCs were eluted from the column by washing with the MACS buffer. The cells were stained with FITC-conjugated rat anti-mouse Sca-1, CD11b, and PE-conjugated rat anti-mouse CD105 (Miltenyi Biotec) at a concentration of 2 *μ*g/mL at 4°C for 30 min. The cells were pelleted, washed twice with PBS, and fixed with 70% EtOH in PBS. Cells were analyzed with flow cytometry using a fluorescence-activated cell sorter (FACS) caliber flow cytometer (B&D Biosciences, Cell Quest software, San Jose, CA, USA).

### 2.5. BMMSCs Differentiation Assays

 The potential for isolated cells to differentiate into osteogenic and adipogenic lineages was examined. For osteogenesis, the cultured cells were incubated in osteogenic conditioning medium as described previously [10]. Briefly, DMEM was supplemented with 10 mM *β*-glycerol phosphate (Sigma-Aldrich Co., St. Louis, MO, USA), 50 *μ*g/mL ascorbate-2-phosphate (Sigma-Aldrich Co.), and 10^−7^ M dexamethasone (Sigma-Aldrich Co.). The culture medium was changed twice per week for up to 3 weeks. The cells were fixed with methanol for 10 min at room temperature and stained with Alizarin red (pH 4.0) for 5 min. Von Kossa staining was used for bone nodule formation. For adipogenesis, the cultured cells were incubated in adipogenic medium DMEM supplemented with 50 *μ*g/mL indomethacin (Sigma-Aldrich Co.), 10^−7^ M dexamethasone, and 50 *μ*g/ mL ascorbate-2-phosphate. The culture medium was changed twice per week for up to 3 weeks. The cells were then fixed in methanol for 45 min and stained with Oil Red O (Sigma-Aldrich Co.).

### 2.6. RNA Isolation and RT-PCR

One milliliter of phenozol was added to 1 × 10^6^ cells, and total RNA was isolated according to the total RNA extraction kit protocol (INTRON Biotechnology, Seoul, Korea). First, strand cDNA synthesis was performed with 5 *μ*g of total RNA using MMLV reverse transcriptase and oligo-dT primer for 1 h at 42°C. Subsequently, PCR amplification was performed using a modified method from that previously described [11]. The sequences of osteocalcin primers were as follows: 5′-GACCATCTTTCTGCTCACTCTG-3′ as forward primer and 5′-GTGATACCATAGATGTTTGTAG-3′ as reverse primer. The sequences of lipoprotein lipase primers were as follows: 5′-GAGGACACTTGTCATCTCATTC-3′ as forward primer and 5′-CCTTCT TATTGGTCAGACTTCC-3′ as reverse primer, whereas for the mouse *β*-actin, 5′-ACCGTGAAAAGATGACCCAG-3′ and 5′-TACGGATGTCAACGTCACAC-3′ were used. PCR products were separated on a 1.5% agarose gel, visualized using ethidium bromide and the i-MAX gel image analysis system (CoreBioSystem, Seoul, Korea), and then analyzed with Alpha Easy FC software (Alpha Innotech, San Leandro, CA, USA).

### 2.7. Cell Viability and Proliferation Assays

Cell proliferation was determined using an MTT assay. We starved mBMMSCs for 24 h and then treated them with ALR (1, 10, and 100 *μ*g/mL). After 24 h, the medium was removed and the cells were incubated with the MTT to measure the metabolic activity. Spectrophotometric analysis at 450 nm was performed using a microtiter plate reader (Molecular Devices, Sunnyvale, CA, USA) to measure the metabolic activity.

### 2.8. Proliferating Cell Nuclear Antigen (PCNA) Detection

For PCNA detection, treated mBMMSCs were fixed and permeabilized in cold methanol, washed three times, and then blocked for 1 h at room temperature in a DMEM-based buffer containing 5% FCS. Samples were washed and incubated overnight with the antibody against PCNA. Following incubation, the cells were washed three more times and incubated for 4 h with goat anti-mouse Alexa 488 antibody (excitation 488 nm, emission 519 nm). For nuclei detection, the cells were incubated for 5 min with propidium iodide (PI) (3.75 *μ*g/mL, excitation 540 nm, emission 630 nm). Imaging was performed using an Olympus BX-61 fluorescent microscope.

### 2.9. Cell Cycle Analysis

For the cell cycle analysis, mBMMSCs in starvation medium were incubated with ALR and various inhibitors for 24 h. The cells were harvested, washed with cold PBS, resuspended in PBS, and fixed in cold ethanol (70%) overnight at 4°C. The cell pellets were washed with ice cooled PBS, collected by centrifugation, and incubated at 37°C for 30 min in a 0.5 *μ*g/mL RNase and PI solution (10 *μ*g/mL). Fluorescence emitted from the PI-DNA complex was quantified after excitation of the fluorescent dye using a FACS (B&D Biosciences, Cell Quest software).

### 2.10. Western Blotting

Growth-arrested preconfluent mBMMSCs were incubated with various inhibitors and ALR for the indicated times. Cells were washed with PBS and lysed in a cell lysis buffer (Promega, Madison, WI, USA). The total cell lysates were separated using SDS-PAGE and were transferred to polyvinyl difluoride (PVDF) membranes (Millipore). The membranes were blocked with 5% skim milk and were washed with PBS-T buffer (PBS in 0.1% Tween 20). The membranes were blotted with primary antibodies: antiphospho-ERK, antiphospho-p38 (Cell Signaling Technology, Beverly, MA, USA), antiphospho-Smad1/5 (Cell Signaling Technology), anti-ERK, anti-p38, anti-Runx2 (Santa Cruz Biotechnology Inc., Santa Cruz, CA, USA), and anti-*β*-tubulin (Santa Cruz Biotechnology). The blots were treated with appropriate secondary antibodies conjugated with horseradish peroxidase (Santa Cruz Biotechnology) and were then visualized by an enhanced chemiluminescence system (Amersham Biosciences, London, UK).

### 2.11. Statistical Analysis

Statistical analysis was performed using GraphPrism 4.0.3 software (GraphPad Software, Inc., San Diego, CA, USA). Data from MTT assay was presented using the mean, standard deviation (SD), and analyzed by the statistical software SPSS, version 12.0 (SPSS, Chicago, IL, USA).

## 3. Result

### 3.1. Detection and Quantification of ALR Indicator Compound Using LC-MS/MS

**Figure 1:**
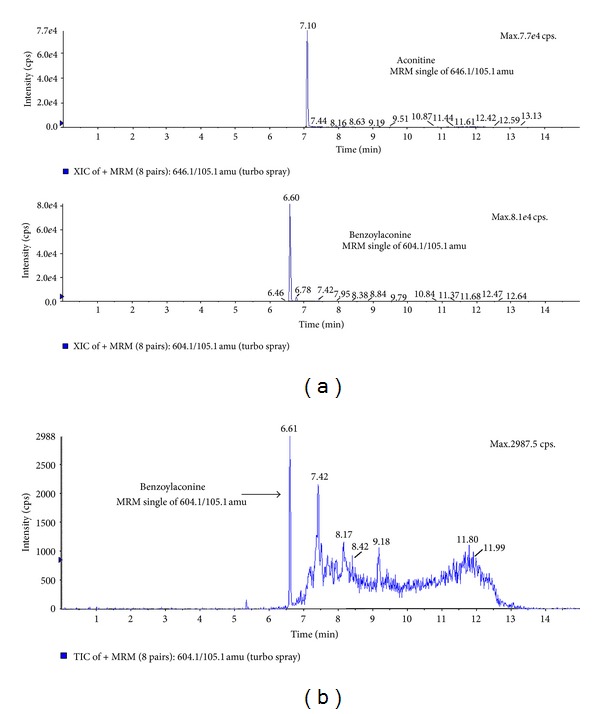
LC-MS/MS chromatograms of analytes in ALR extract. (a) Reference standards, aconitine, and benzoylaconine of ALR were detected at corresponding retention times. (b) Detection of indicative compound from 100 *μ*g/mL of ALR extract. LC/MS/MS analysis showed that ALR extract contained benzoylaconine.

 To identify and confirm the indicative compounds of ALR, 100 *μ*g of lyophilized ALR was analyzed using an LC/MS/MS method. The MRM acquisition for benzoylaconine was detected at 105.1 with a retention time at 6.62 min (Figure 1(a)), but aconitine was not detected from 100 *μ*g/mL of ALR extract. The result showed that 100 *μ*g/mL of ALR extract contained 1.43 ng/mL of benzoylaconine (Figure 1(b)). 

### 3.2. mBMMSCs Isolation and Characterization

**Figure 2:**
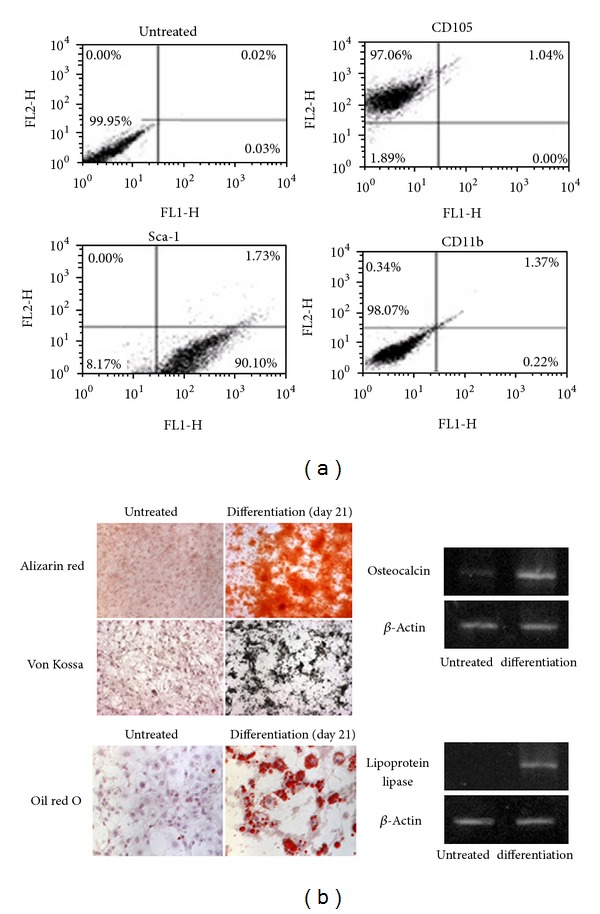
mBMMSC isolation and characterization using FACS analysis of surface markers and multilineage mesenchymal stem cell differentiation. (a) FACS analysis using positive (CD105, Sca-1) and negative (CD11b) selections. (b) The differentiation of mBMMSCs cells examined at 21 days following treatment shows the stimulation of osteogenesis and adipogenesis. Left panel: cells were stained with Alizarin red and von Kossa to identify osteogenic differentiation. To recognize adipogenic differentiation, lipid vesicles were stained with Oil Red: magnification, 40x. Right panel: mRNA levels of osteoblast- and adipocyte-specific markers such as osteocalcin and lipoprotein lipase were analyzed using RT-PCR.

To isolate and characterize the mBMMSCs, magnetic bead separation was carried out as described above. After negative and positive selections of bone marrow-derived cells with CD45 and Sca-1, collected cells were immunophenotyped for the detection of different mBMMSC surface antigens. Cells were positively stained with FITC-conjugated rat anti-mouse Sca-1 and PE-conjugated rat anti-mouse CD105. Cells were also negatively stained with FITC-conjugated rat anti-mouse CD11b. The expressions of CD105, CD11b, and Sca-1 were characterized using FACS analysis. FACS analysis revealed that 97.06 and 90.10% of the selected cells expressed CD105 and Sca-1, respectively, (Figure 2(a)). Analysis of CD11b expression in cultured cells indicated that the level of its expression was 0.22%, which means that there was no contamination of hematopoietic cell lineage (Figure 2(a)).

To confirm the isolation of mBMMSCs, we examined the differentiation potential of mBMMSCs into multiple cell lineages. The BMMSCs were readily differentiated into osteocytes and adipocytes by culturing in appropriate induction media. In osteogenic cultures, nodule-like structures, which were stained with Alizarin red, were observed after 3 weeks of induction (Figure 2(b)). For the detection of calcium-phosphate deposits, a typical phenomenon for bone cells, osteogenic cultures were stained by the von Kossa method. As a result, cells showed calcium phosphate deposits equivalent to those observed in osteoblast cells. Likewise, after 2 to 3 weeks of mBMMSCs culture, the adipocytes were stained with Oil Red O. Adipose droplets were visible in adipogenic inductive medium (Figure 2(b)). 

To further demonstrate the differentiation potential of mBMMSCs into multiple cell lineages, we analyzed the expression of osteoblast- and adipocyte-specific markers using RT-PCR. Expression levels of osteocalcin, an osteoblast specific marker, were elevated, and the lipoprotein lipase gene, an adipocyte-specific marker, was expressed in differentiation cells following 21 days of induction.

### 3.3. ALR Enhances mBMMSCs Proliferation through Progression of the Cell Cycle

**Figure 3:**
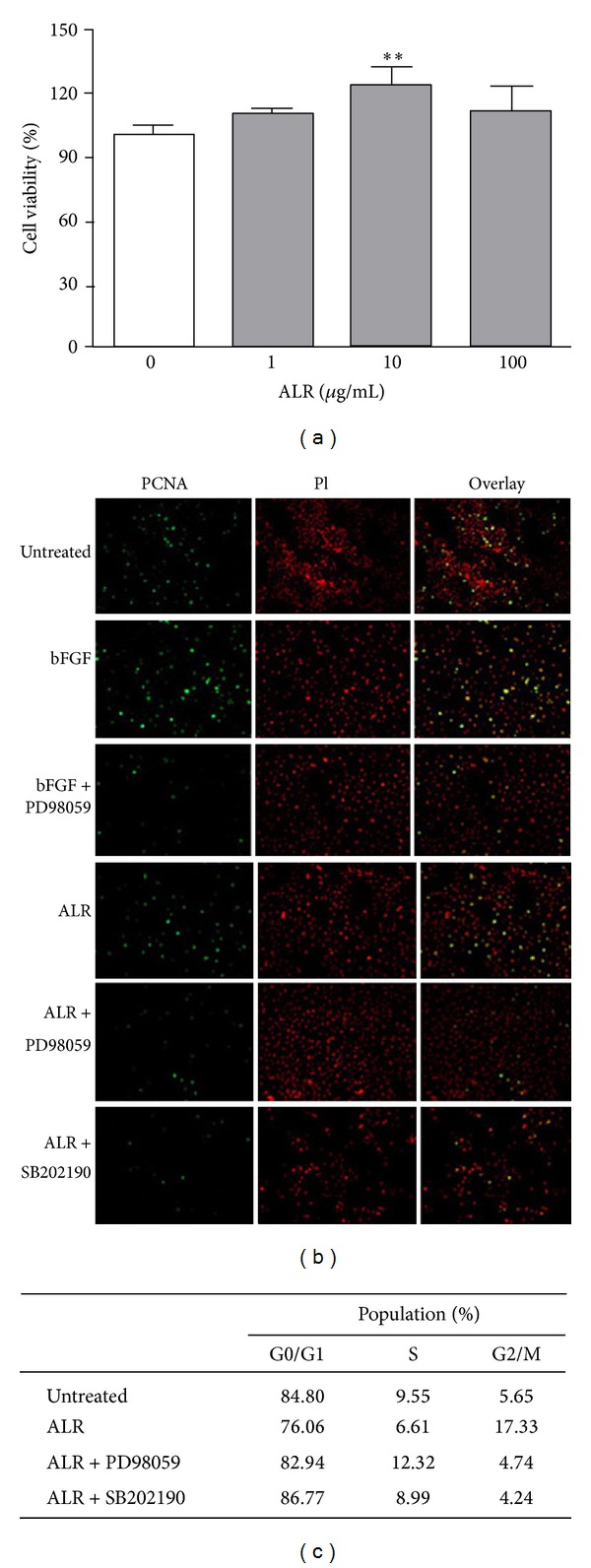
(a) The effect of ALR on the growth of mBMMSCs. “Normal” represents PBS-treated cells, columns represent the mean ± SD (*n* = 3), and * indicates that the mean is significantly different from the control value (**P < 0.01). (b) Proliferating cell nuclear antigen (PCNA) immunoreactivity measurements. Cells are stained with either anti-PCNA antibodies (green) or propidium iodide (red) for nuclear detection. Overlay images of both stains are also presented. bFGF was used as a positive control. All images were obtained using 20x magnification in an Olympus BX-61 fluorescent microscope. (c) The effect of ALR on the progression of mBMMSCs cell cycle. Results are the mean of three independent experiments.

The effects of ALR on the proliferation of mBMMSCs were determined from cell growth kinetics with an MTT assay measuring the metabolic activity of viable cells. Growth-arrested mBMMSCs cultured in a starvation medium for 24 h prior to the experiment were incubated for 24 h with ALR. ALR activated cell proliferation; specifically, 10 *μ*g/mL ALR-treated cells increased by 122.24 ±8.78% when compared with untreated cells (*P* < 0.01, Figure 3(a)). This suggests that ALR enhances the proliferation rate of mBMMSCs.

To further confirm the proliferating effects of ALR using cell cycle analysis, we examined the activity of PCNA, a protein that participates in DNA replication. Cells were grown in a medium treated with ALR (100 *μ*g/mL, 24 h) in the presence or absence of SB202190 and PD98059, inhibitors of p38, and ERK1/2, respectively. Cells were then incubated with the fluorescent DNA binding dye, PI, for nuclear staining (red) and with an antibody directed against PCNA (green). ALR increased the level of PCNA staining compared to untreated cells, indicating that ALR activates BMMSC proliferation as observed in bFGF-treated cells (Figure 3(b)). Treatment with bFGF as a positive control is known to induce cell proliferation by activating the ERK1/2 pathway in many cell types. Pretreatment with SB202190 and PD98059 decreased the expression level of PCNA compared to cells treated with ALR alone. Taken together, from the FACS analysis, ALR promoted proliferation (G2/M) in mBMMSCs, whereas untreated cells remained in the G1 phase of the cell cycle (Figure 3(c)). Pre-treatment of mBMMSCs with PD98059 impaired this proliferation, inducing a G1 arrest as was also seen in treatment with SB202190. These results suggest that ALR enhances mBMMSC proliferation throughout the cell cycle by promoting pathways such as ERK1/2 and p38 cascades, which appear to be implicated in the G1 to S and S to G2/M switches.

### 3.4. ALR Activated mBMMSC Proliferation through the ERK1/2 Pathway

**Figure 4:**
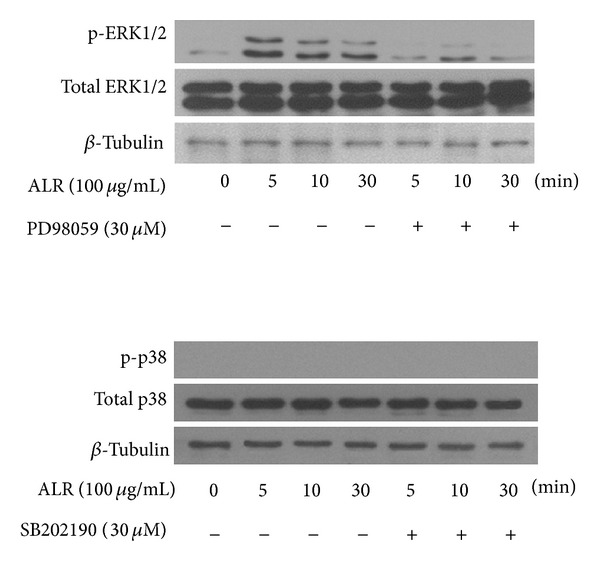
Immunoblots of ERKs and p38 detected with specific antibodies p-ERK1/2, p-p38, total ERK, and total p38 after ALR treatment with *β*-tubulin used as an internal control. mBMMSCs were incubated with ALR (100 *μ*g/mL) over time in the presence or absence of inhibitors, SB202190 (30 *μ*M, 2 h), and PD98059 (30 *μ*M, 2 h).

To elucidate the signaling events triggered by ALR treatment in mBMMSCs, western blot analyses were carried out. Growth-arrested mBMMSCs were incubated with ALR for 5, 10, and 30 min in the presence of PD98059 (30 *μ*M, 2 h) prior to their incubation. Whole cell lysates were then immunoblotted using phospho-ERK1/2 antibodies. As shown in Figure 4, ALR treatment increased phosphorylation of ERK1/2, while ALR treatment in the presence of inhibitors led to decreased phosphorylation of ERK1/2. At the same time, growth-arrested mBMMSCs were incubated with ALR for 5, 10, and 30 min in the presence of SB202190 (30 *μ*M, 2 h) prior to their incubation. Whole cell lysates were immunoblotted using total p38 and phospho-p38 antibodies, but there was no change in the expression level of either protein (Figure 4). These results suggest that ALR activates the proliferation of mBMMSCs through the ERK1/2 pathway.

### 3.5. ALR Promoted Differentiation of mBMMSCs into the Osteogenic Lineage

**Figure 5:**
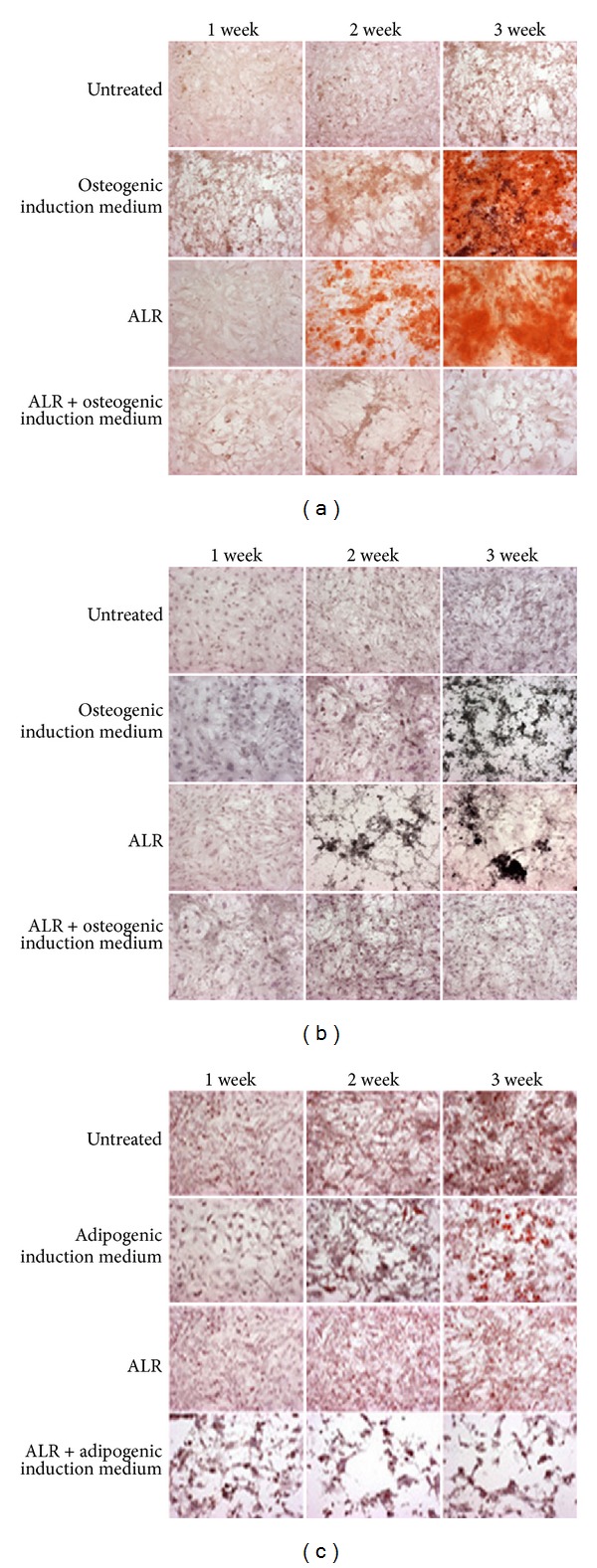
*In vitro* osteogenic and adipogenic differentiations of mBMMSCs by ALR. (a) Osteogenic cultures with Alizarin red staining for the detection of nodule-like structures; (b) osteogenic cultures stained with von Kossa for the detection of calcium-phosphate deposits; (c) adipogenic cultures stained with Oil Red for the detection of adipose droplets. Original magnification is 20x.

An important feature of mBMMSCs is their ability to differentiate into osteoblasts, chondroblasts, and adipocytes. To examine whether ALR affected the osteogenic and adipogenic differentiation capacities of the cells, mBMMSCs were treated with ALR (100 *μ*g/mL) for 3 weeks and were examined once per week. Both the Alizarin red and von Kossa assays showed a strong induction of mBMMSCs into the osteogenic lineage by ALR treatment. As shown in Figures 5(a) and 5(b), ALR induced osteogenic differentiation after 2 weeks of treatment, whereas induction by osteogenic medium did not begin for 3 weeks. In the adipogenic differentiation assay, lipid vacuoles were stained with Oil Red. Induction showed adipogenic differentiation at the end of 3 weeks; however, ALR treatment did not induce adipogenic lineage differentiation even after 3 weeks.

### 3.6. Signaling Pathway for ALR Stimulated Osteogenic Differentiation

**Figure 6:**
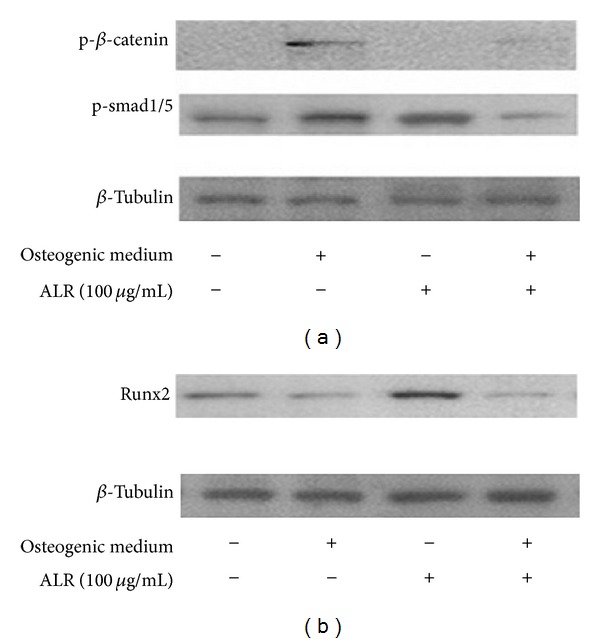
Immunoblots of p-Smad1/5, p-*β*-catenin, and total Runx2 after ALR treatment on mBMMSCs. *β*-tubulin was used as internal control. (a) Immunoblots conducted for the detection of Smad1/5 and p-*β*-catenin phosphorylation of using extract of mBMMSCs; (b) immunoblots carried out for the detection of Runx2on expression in mBMMSCs extract treated with ALR (100 *μ*g/mL) or osteogenic induction medium.

To identify the signaling pathway that triggers the osteogenic differentiation of mBMMSCs by ALR, BMP-2/Smad and Wnt pathways were examined. For this, mBMMSCs were treated with either ALR (100 *μ*g/mL) or osteogenic induction medium for 21 days. The BMP-2/Smad signal pathway was activated and Runx2 protein expression was increased by ALR treatment in osteogenic differentiation. Whole cell lysates were immunoblotted using phospho-Smad1/5, phospho-*β*-catenin, and Runx2 antibodies. As a result (Figure 6(a)), ALR treatment led to the increased phosphorylation of Smad1/5, while treatment with both ALR and osteogenic induction medium had no effect on the phosphorylation of Smad1/5. Phosphorylation of *β*-catenin was also increased by osteogenic induction medium treatment. However, ALR treatment decreased the phosphorylation of *β*-catenin. Subsequently, Runx2 protein levels were increased by ALR treatment and were decreased when treated with both ALR and osteogenic induction medium, when compared with untreated mBMMSCs (Figure 6(b)). These results suggest that ALR activates osteogenic differentiation through BMP-2/Smad-dependent Runx2 signaling pathway. 

## 4. Discussion

The aim of this study was to investigate the effect of ALR on mBMMSC proliferation and differentiation and to elucidate the signaling pathways involved in these processes. ALR has been widely used for the improvement of symptoms such as, heart failure, inflammation, pain and diarrhea for thousands of years in Korea, China and Japan, but the toxicity of the ALR has been the subject of controversy in safety as medicinal herb. Major toxic ingredients were identified as some kinds of diester diterpenoid-type Aconitum alkaloids such as aconitine and mesaconitine [12]. Some processing techniques such as pressure-steaming were known to reduce the toxicity of Aconitum alkaloids, because alkaloids can hydrolyze the highly toxic diester-diterpene aconitum alkaloids to compounds of much lower toxicity such as benzoylaconine, benzoylmesaconine, benzoylhypaconine, and aconine [13]. LC/MS/MS results in this study showed that 100 *μ*g/mL of ALR extract contained about 1.43 ng/mL of benzoylaconine, one of an indicative compound of ALR. However, aconitine was not detected from ALR extract. This could be interpreted in part by the hydrolysis of aconitine into other compounds during ALR extract preparation which includes the boiling step to extract active compounds. At least in cellular level, this was further confirmed through cell viability and cell cycle progression analysis. As shown in Figure 3, ALR did not show any cytotoxicity on mBMMSC growth and proliferation.

Bone marrow-derived adherent cell layers are composed of heterogeneous cells, including fibroblasts, hematopoietic progenitor cells, macrophages, endothelial cells, and adipocytes [9]. The purification of mBMMSCs has been more difficult than that of other species due to lack of specific surface markers for MSCs and the unwanted growth of non-MSCs in cell cultures [7, 9]. A more homogenous population of MSCs can be acquired after the third passage with frequent medium changes, in the absence of molecular markers [14, 15]. For successful MSC transplantation, it is important to obtain an established homogeneous MSC purification tool using surface markers [16]. 

Bone marrow was harvested from C57BL/c and maintained as described in the Materials and Methods section. During the *in vitro* expansion of mBMMSCs, cell morphology changed gradually from a fibroblast-like spindle shape to more flattened- and enlarged-shaped cells that were more homogeneous. The mouse mesenchymal stem cells expressed CD34, CD44, Sca-1, and Vcam-1 antigens (markers) but not CD11b and CD45. Therefore, the magnetic-activated cell sorting (MACS) method was used for the selection of mBMMSCs using positive (Sca1) and negative surface (CD11b) markers at passage 3, followed by confirmation with Sca-1-positive and CD11b-negative cells (Figure 2(a)) [9, 17]. The differentiation of mBMMSCs was confirmed by staining methods for both osteogenic and adipogenic lineage differentiations (Figure 2(b)).

MSCs can be applied in regenerative therapies such as the treatment of graft-versus-host disease and in cell therapies for their lack of strong immunogenicity [18]. Although MSCs are an appropriate tool in cell-based therapies, clinical application of MSCs is currently limited by the low number MSCs available for transplantation [19]. Thus, many researchers have been searching for a way to improve cell proliferation and/or differentiation by experimenting with various types of growth factors, despite the obscure molecular mechanisms of cell expansion. In this study, ALR promoted the proliferation of mBMMSCs. To detect mBMMSC proliferation by ALR treatment, we examined PCNA, a protein that participates in DNA replication. Results showed that ALR increased the level of positively stained PCNA when compared to that of untreated cells. In addition, the G1-to-S phase progression mechanism was found to be essential for cell cycle regulation. We performed a cell cycle analysis to examine the cell cycle transition in the ALR-treated proliferating cells. Results showed that ALR-treated cells expressed elevated G2/M progression compared to untreated cells. 

To further understand ALR's effect on the cell cycle progression of mBMMSCs, the signaling pathways responsible for cell cycle progression were investigated. MAPK pathways are a continuative protein kinase cascade that regulates cell proliferation, differentiation, inflammatory response, and apoptotic cell death [20]. The ERK pathway (A-Raf, B-Raf, Raf-1 MEK1, and 2 ERK1/2) is activated by mitogens and growth factors, and activated ERK is translocated to the nucleus. Translocated ERK regulates transcription factors, changing gene expression to promote growth, differentiation, or mitosis. The p38 pathway is stimulated predominately by UV irradiation, heat shock, high osmotic stress, lipopolysaccharide, protein synthesis inhibitors, proinflammatory cytokines environmental stress, and inflammatory cytokines [21, 22]. In our results, ALR activated ERK1/2 phosphorylation in mBMMSCs but did not activate p38. These results strongly suggest that ALR enhances the proliferation of mBMMSCs though activating ERK1/2 pathway. 

In terms of differentiation, ALR induced mBMMSCs differentiation into the osteogenic lineage, but not into the adipogenic lineage. Glucocorticoids, such as dexamethasone, are known to promote osteoblastic differentiation *in vitro* and to regulate the expression of osteoblast typifying genes such as Runx2, ALP, and osteocalcin in MSCs. Also, endogenous glucocorticoids have been found to differentiate MSCs through osteogenesis in mouse bone marrow [23]. Our results demonstrate that ALR promoted the differentiation of mBMMSCs into osteogenic lineage 2 weeks following treatment, as opposed to the osteogenic induction medium (including dexamethasone), which did not lead to differentiation for 3 weeks. 

The regulation of osteoblast differentiation plays an important role in BMP-2 signaling and Wnt signaling pathways. In BMP-2 signaling, BMP-2 activates Smad1/5 and Smad4 and induces Runx2 expression. Subsequently, gene expression related to osteogenesis differentiation is promoted, as observed in the homeobox gene, msh homeobox homologue 2, or Osterix [24]. Wnt signaling has two different pathways, canonical and noncanonical pathway. The Wnt signal is transduced through Frizzled and Lrp5/6 and stabilizes *β*-catenin for translocation into the nucleus and for regulation of the expression of target genes. Wnt1, Wnt2, and Wnt3 induce alkaline phosphatase activity in mesenchymal cell lines. It has been reported that canonical Wnt *β*-catenin and BMP signaling induce osteogenic differentiation [24]. According to our results related with signaling pathways involved in osteogenic differentiation, ALR treatment for 3 weeks enhanced the phosphorylation of Smad1/5 without any effects on the Wnt/*β*-catenin pathway. Overall, Runx2 expression in ALR-treated cells increased in undifferentiated cells. However, the expression level of Runx2 was reduced in dexamethasone-induced osteogenic differentiation, and *β*-catenin expression was activated by dexamethasone. Similarly, the cotreatment of mBMMSCs with ALR and osteogenic induction medium showed decreased expression of Runx2. 

Runx2, also known as Cbfa1, Osf2, AML3, and PEBP2*γ*A, is a critical transcriptional factor in osteoblast differentiation and bone formation [25]. However, in terms of the signaling pathways involved in Runx2-mediated osteogenic differentiation, it has been suggested that FHL2, an LIM-domain protein with 4.5 LIM domains, interacts with *β*-catenin to increase Runx2 expression and to induce the osteogenic differentiation of mesenchymal stem cell [26]. However, Phillips et al. demonstrated that glucocorticoid-induced osteogenic differentiation decreased the phosphoserine levels associated with Runx2 and that exogenous Runx2 can antagonize the effect of dexamethasone [23, 25]. Thus, one explanation for our results is an antagonistic effect of dexamethasone and ALR-cotreated cells in regard to Runx2 expression. 

In conclusion, this study demonstrated that after 2 weeks of treatment, ALR induced osteogenic differentiation and increased both cell proliferation and osteogenic differentiation in mBMMSCs, processes mediated by the BMP-2/Smad-dependent Runx2 signal pathway. Although the molecular mechanism of ALR action in stem cell regulation, growth, and differentiation requires further study, this study is an important step in overcoming the limitations to stem cell therapy in clinical uses such as transplantation, *in vitro* expansion, and endogenous induction of MSC proliferation and differentiation. Moreover, ALR may be considered as a medicinal herb for the treatment of bone-related diseases such as osteoporosis and osteopenia.
